# Exploring the Determinants of Repeated-Sprint Ability in Adult Women Soccer Players

**DOI:** 10.3390/ijerph18094595

**Published:** 2021-04-26

**Authors:** Lillian Gonçalves, Filipe Manuel Clemente, Joel Ignacio Barrera, Hugo Sarmento, Francisco Tomás González-Fernández, Markel Rico-González, José María Cancela Carral

**Affiliations:** 1 Faculty of Educational Sciences and Sports Sciences, University of Vigo, 36005 Pontevedra, Spain; chemacc@uvigo.es; 2 Escola Superior Desporto e Lazer, Instituto Politécnico de Viana do Castelo, Rua Escola Industrial e Comercial de Nun’Álvares, 4900-347 Viana do Castelo, Portugal; filipe.clemente5@gmail.com; 3 Instituto de Telecomunicações, Delegação da Covilhã, 1049-001 Lisboa, Portugal; 4 University of Coimbra, Research Unit for Sport and Physical Activity, Faculty of Sport Sciences and Physical Education, 3004-531 Coimbra, Portugal; jibarrera@outlook.es (J.I.B.); hg.sarmento@gmail.com (H.S.); 5 Department of Physical Activity and Sport Sciences, Pontifical University of Comillas (Centro de Estudios Superiores Alberta Giménez), 07013 Palma, Spain; francis.gonzalez.fernandez@gmail.com; 6 BIOVETMED & SPORTSCI Research Group, University of Murcia, 30720 San Javier, Spain; markeluniv@gmail.com; 7 Department of Physical Education and Sport, University of the Basque Country, UPV-EHU, Lasarte 71, 01007 Vitoria-Gasteiz, Spain

**Keywords:** football, athletic performance, anaerobic, aerobic, sports training

## Abstract

This study aimed to explore the main determinants of repeated-sprint ability (RSA) in women soccer players considering aerobic capacity, sprinting performance, change-of-direction, vertical height jump, and hip adductor/abductor isometric strength. Twenty-two women soccer players from the same team participating in the first Portuguese league were observed. Fitness assessments were performed three times during a 22-week cohort period. The following assessments were made: (i) hip abductor and adductor strength, (ii) squat and countermovement jump (height), (iii) change-of-direction test, (iv) linear sprinting at 10- and 30-m, (v) RSA test, and (vi) Yo-Yo intermittent recovery test level 1. Positive moderate correlations were found between peak minimum RSA and adductor and abductor strength (r = 0.51, *p* < 0.02 and r = 0.54, *p* < 0.01, respectively). Positive moderate correlations were also found between peak maximum RSA and adductor and abductor strength (r = 0.55, *p* < 0.02 and r = 0.46, *p* < 0.01, respectively). Lastly, a moderate negative correlation was found between fatigue index in RSA and YYIR1 test performance (r = −0.62, *p* < 0.004). In conclusion, abductor and adductor isometric strength-based coadjutant training programs, together with a high degree of aerobic endurance, may be suitable for inducing RSA in female soccer players.

## 1. Introduction

Soccer is a team sport practiced by many athletes throughout the world, with an estimated 4–26 million female participants [1,2,3,4] and approximately 238 million male participants [5]. The number of female soccer players has increased in the last years in approximately 50% considering the last report of FIFA [3,6]. Due to the challenges associated with this rapid increase in the number of participants, it is important to better understand the characteristics of these players, their physiological/physical demands, and their training processes [1,2,7].

As an intermittent exercise, a women’s soccer match involves activities with different intensities, such as walking, jogging, moderate running, high-intensity running, and sprinting [8,9,10]. It is well-known that low-intensity movements are predominant during women’s matches [9,11,12], although high-intensity activities are also considered important components of physical performance, and they are often crucial to the outcomes of matches because they are associated with offensive attacks [12,13]. Usually, women soccer players cover between 8.5 and 11 km in a match, of which 1.5–1.8 km are spent performing high-speed running and from 14.9 to 460 m are spent sprinting [4,9,10,14,15].

To support the demands of the match, a proper fitness status should be sustained. As an example, in previous research on women soccer players, a strong correlation was observed between Yo-Yo intermittent recovery test performance and the amount of high-intensity running performed in games [9,16]. Additionally, a strong correlation between sprinting skills and high-intensity performance was found in a previous study [2]. In fact, many decisive phases during a soccer match require players to exercise at a high intensity [17]. Therefore, the ability of a soccer player to recover and to reproduce their performance in subsequent sprints is a vital fitness condition [10]. In the particular case of elite level, the intermittent high-intensity endurance and the ability to repeatedly sprint in short time intervals (RSA) are considered relevant fitness conditions for competitive soccer players [18,19,20,21,22].

As a multifactorial factor, the RSA can be influenced by anaerobic and aerobic metabolism [18,23,24,25]. From a physiological perspective, RSA is a complex quality that is correlated with motor unit activation and is essential to achieving maximal sprint speed and oxidate capacity for phosphocreatine (PCr) recovery and hydrogen (H+) buffering to provide the ability to repeated sprints [26]. Following the same line of thinking, other authors have shown that better sprinters use more of their accessible PCr stores than weaker sprinters [27]. This idea could be related to the strong relationship between PCr resynthesis and power output recovery following 30-s sprints [27,28].

The RSA test simulates intermittent exercise and identifies a player’s capacity to maintain maximal effort and recovery during multiple successive high-speed running or sprinting efforts [20,29,30,31]. Therefore, RSA is an essential factor for determining success in soccer, alongside other qualities like technical and tactical skills, strength, explosive power, speed, and endurance [26,32]. When RSA is compared with aerobic capacities, it was concluded that players with a higher aerobic capacity and faster oxygen kinetics recover faster after high-intensity exercise [29]. These athletes also exhibited better overall RSA performance and recovery performance during the RSA test [29]. Another study showed that subjects with a higher maximal oxygen consumption (VO_2_max) value present smaller sprint decrements, suggesting that VO_2_max contributes to maintaining performance during repeated-sprint efforts [27].

Beside the metabolic perspective that supports RSA, physical capacities also play a determinant role in RSA. As example, a well-developed neuromuscular system allows a better activation of motor unit [26], while lower-limb strength and power support the acceleration and the maximal speed in the first repetitions and aerobic capacity sustain the performance over the last sprints [33]. The efficiency of RSA could also depend on the player’s agility, as this factor is known to be correlated with linear sprint ability [34,35,36].

The ability to perform repeated sprints while requiring minimal recovery periods between efforts (RSA) appears to be an important aspect of field-based team sport [37]. However, it is difficult to understand which determinants are related to RSA. Thus, some doubts and non-consensual evidence remain in this regard in women’s soccer. For that reason, it is important to identify which physical capacities could explain RSA in women’s soccer. Such identification may help coaches define better strategies for improving RSA. Therefore, the purpose of this study was to analyze the determinants of RSA based on aerobic performance, linear sprinting and change-of-direction, vertical height jump, and abductor and adductor isometric strength. We hypothesize that strength and power will be determinants for maximum and minimum peak power RSA, while aerobic performance will be determinant for sustaining the performance (fatigue index) [33].

## 2. Materials and Methods

### 2.1. Experimental Approach to the Study

**Figure 1:**
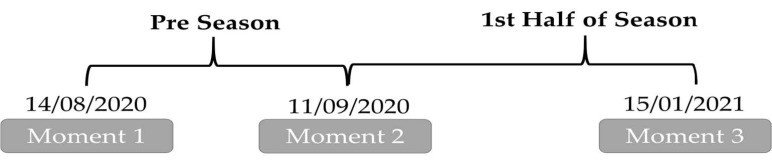
Timeline of the study.

This study followed an observational analytic cohort design. The period of observation was 22 consectutive weeks. Fitness assessment were performed three times during the cohort (Figure 1). Between the first and second assessment occurred 4 weeks (pre-season) and between the second and third assessment 18 weeks (end of the first half of the season). The aim was to explore determinants of RSA based on the measures of aerobic capacity, sprinting performance, change-of-direction, vertical height jump and hip adductor/abductor isometric strength. From the initial twenty-five participants, twenty-two remained. Three were excluded based on the fact that did not participated in all the assessments.

### 2.2. Participants

Twenty-two women soccer players from the same team participating in the first Portuguese league were observed during the study. In the beginning of the season, the participants presented a mean age of 22.7 ± 5.21 years old, 162.51 ± 7.08 cm of height, 59.1 ± 9.50 kg of body mass. In the second assessment the mean of body mass was 59.01 ± 9.31 kg and in the third evaluation the mean body mass was 61.10 ± 9.94 kg. The eligibility criteria for including in the final sample was: (i) participants were assessed in the three moments of the cohort; (ii) participants participated in, at least, 85% of the total number of training sessions during the cohort; (iii) players had injuries or illness no longer than 4 consecutive weeks; and (iv) players should had a minimum of two years of experience to volunteered for this study. Among the included participants, three were goalkeepers, four were external defenders, four were central defenders, six were midfielders, and five were attackers. The team had three training sessions per week plus an official match in the weekends. Before the cohort begin, all the players were informed about the study design and procedures. After that, each player signed an informed consent. The study was approved by the local university (code: CTC-ESDL-CE001-2021) and followed the ethical standards of Declaration of Helsinki for the study in humans. 

### 2.3. Data Collection

In the three moments of assessment, the tests were made always at the same hour (7:30 p.m.) and days of the week, with a rest period of 48 h (considering the last match/training). Additionally, the assessments two and three were preceded by the same type of microcycle. In each moment of the assessment, the tests were split over three days (interspaced by 24 h). In the first training session of the week players were tested for their anthropometry and hip adductor and abductor strength. In the second training session it was assessed the vertical jump, changes of direction and linear speed. In the third session it were applied the repeated sprint ability test and the Yo-Yo intermittent recovery test. Before the first assessment of each day, a standard warm-up protocol was implemented, by group of players, since they were organized in groups of three to have the same duration between the end of warm-up and beginning of the test. All the players followed the same order. Between tests, there was a minimum of 3 min of rest. The anthropometry, abductor and adductor strength and squat and countermovement jump were performed in a private room, with a stable temperature of 23 °C and relative humidity of 55%. The sprinting tests, RSA test and the Yo-Yo intermittent recovery test were executed in a synthetic turf with a mean temperature of 19.5 ± 3.4 °C and relative humidity of 63 ± 4%. No raining conditions occurred in the assessments. 

#### 2.3.1. Anthropometry

There was collected the height and body mass in the three moments of evaluation, at the same hour and at the same day of the week. The evaluation of the height was executed by using the stadiometer (SECA 213, Birmingham, UK), players were asked to remove shoes and other accessories that influence the assessment, they also should be in a vertical and immobile position, with the arms extended along the body and keep a fixed stare, straight ahead and in an upright position. The evaluation of the body mass was executed with a digital balance (SECA 869, Birmingham, UK), it was asked to the players to be barefoot and only in light clothing. For each measure, only one measured was collected.

#### 2.3.2. Hip Adductor and Abductor Isometric Strength

Hip adductor and abductor isometric strength measurement was tested with the dynamometer (Smart Groin Trainer, Neuro excellence, Braga, Portugal). The dynamometer was positioned in the thigh area. Players were asked to lie down in the supine position, with 45° of hip flexion and around 90° of knee flexion [38]. Players were instructed to execute the maximum squeeze in accordance with a previous study [38], although with changes to 20 s for the present protocol. Three trials were made for abductor and adductor, with 10 s of rest between trials. Abductor were tested first (all the trials) and then adductor (all the trials). The highest strength in kilograms were extracted as the main outcome. The best score among trials was obtained for the data treatment.

#### 2.3.3. Squat and Countermovement Jump

The squat and countermovement jumps were performed. The squat jump (SJ) consisted in standing with the knees at 90 degrees, as the position of squat, with no movement, hand in the waist, with no help of the upper limbs the player should jump and extend the legs, falling in the same place. The players waited 3 s in squat position before each jump. The countermovement jump (CMJ) started in standing position with the hands in the waist, being realized with the flexion of the legs and immediately the extension with the jump, the legs will be in extension and they will fall in the same place. For each movement, three trials were executed, with a rest period of 30 s between. The SJ and CMJ were tested with an optical measurement system consisting of a transmitting and receiving bar (Optojump, Microgate, Bolzano, Italy). The Optojump allows a repeatable measurement of flight time as confirmed in a reliability experiment with an intraclass correlation test of 0.95 [39]. The outcome extracted in each trial was the jump height (cm). For each movement, it was considered the highest jump for data treatment. 

#### 2.3.4. Change-of-Direction Test

Agility was assess by using the test zig-zag 20 m [40], this test consisted in four sections of 5 m each set out at 100°. The time was recorded using photocells timing gates (Photocells, Brower Timing System, UT, USA), with resolution of 1 thousandth of seconds. Typical error of the Photocells was between 0.04 and 0.06 (s), while the smallest worthwhile change was between 0.11 and 0.17 (s) [41]. This test was performed in the fields, before the training session. Subjects performed three trials of the test, with 3 min of rest between all trials and tests. The outcome extracted was the best time (lowest time in seconds) considering the trials.

#### 2.3.5. Linear Sprinting

Linear Sprint was assessed over 10-m and 30-m using photocell timing gates (Photocells, Brower Timing System, USA), with resolution of 1 thousandth of seconds. The participants started 0.5 m behind the initial timing gate in a two point split stance and were instructed to set off in their own time and run at the maximal speed until the last gate. Each participant performed three trials at maximal effort. The outcome extracted was the time (seconds) for completing the run. The best score in each running distance was considered for the data treatment. 

#### 2.3.6. Running Anaerobic Sprint test

The protocol used for testing the RSA consisted in 35 linear meters (no change-of-direction), performed six times and with a recovery time between efforts of 10 s [42]. The participants started their sprint 0.5 m behind the starting timing gate. Photocell timing gate (Photocells, Brower Timing System, UT, USA), with resolution of 1 thousandth of seconds were positioned in the beginning and at the end lines to record the time of each sprint effort. The time (seconds) for each trial was collected. After that, the minimum and maximum peak power was determined by using the equation [43] $\mathrm{Power}=\frac{\mathrm{Body}\;\mathrm{mass}\times {\mathrm{Distance}}^{2}}{{\mathrm{Time}}^{3}}$, as well as the fatigue index used the following equation [43] $\mathrm{Fatigue}\;\mathrm{index}=\frac{\max_{\mathrm{power}}-\min_{\mathrm{power}}}{\mathrm{Sum}\;\mathrm{of}\;6\;\mathrm{sprints}\;\left(s\right)}$.

#### 2.3.7. Yo-Yo Intermittent Recovery Test—Level 1

The Yo-Yo IR1 test consisted of repeated 20-m runs back and forth between two markers with a progressive increase in speed, which was regulated by an audio player. Between each 40-m run, the athlete recovered with 10 s of jogging (shuttle runs of 2 × 5 m). Yo-yo level 1 starts at 10 km/h and level 2 at 13 km/h, with both levels progressively increasing in speed throughout the test. The test was completed when the athlete reached voluntary exhaustion or failed to maintain her running pace in synchrony with the audio recording. The number of completed levels and shuttles and the total distance covered were recorded at the end of the test. The total distance (meters) was extracted as the outcome. The maximal oxygen Uptake (VO_2_max in mL/min/kg) was estimated by the next equation [44]: ${\mathrm{VO}}_{2}\max=\mathrm{final}\;\mathrm{distance}\;\left(m\right)\times 0.0084+36.4$.

### 2.4. Statistical Analysis

For the treatment of the data, we use adequate statistical methods to calculate percentages and central and dispersion parameters (arithmetic mean and standard deviation). Descriptive statistics were calculated for each variable (See Table 1, for more information). In ADD and ABD two subjects missed the data collection, and they were excluded from the item analysis. Similarly occurred with one participant in YYIRT. Before any parametric statistical analysis was performed, the assumption of normality was tested with the Kolmogorov–Smirnov test on each variable. The changes over the season were determined by a one-way ANOVA with repeated measures. Significant main effects were subsequently analyzed using a Bonferroni post hoc test. Effect size is indicated with partial eta squared for Fs. To interpret the magnitude of the eta squared we adopted the following criteria: η^2^ = 0.02, small; η^2^ = 0.06, medium; and η^2^ = 0.14 large. Pearson correlation coefficient r was used to examine the relationship between RSA (Pmax, Pmin, and Fatigue index) and the remaining variables (ADDs, ABDs, SJ, CMJ, 10 and 30 m sprint, COD and YYIRT1). To interpret the magnitude of these correlations we adopted the following criteria: r ≤ 0.1, trivial; 0.1 < r ≤ 0.3, small; 0.3 < r ≤ 0.5, moderate; 0.5 < r ≤ 0.7, large; 0.7 < r ≤ 0.9, very large; and r > 0.9, almost perfect [45]. Confidence intervals (95% CI) were calculated for each correlation. Multiple regression analysis was used to model the prediction of RSA from remaining variables. In this regression analysis, were examined separately all variables. Data were analyzed using software Statistica (version 10.0; Statsoft, Inc., Tulsa, OK, USA). 

## 3. Results

Descriptive statistics were calculated for each variable (See Table 1, for more information). 

Different repeated measures ANOVAs with participants’ mean ADDs, ABDs, 10 m, 30 m, COD and FI, did not revealed any effect of moment F (1.16) = 0.00080, *p* = 0.97, η^2^ = 0.001, F (1.16) = 0.00063, *p* = 0.98, η^2^ = 0.001, F (2.28) = 1.39, *p* = 0.26, η^2^ = 0.09, F (2.28) = 2.81, *p* = 0.07, η^2^ = 0.16, F (2.26) = 1.18, *p* = 0.32, η^2^ = 0.08, and F (2.26) = 0.99, *p* = 0.38, η^2^ = 0.07, respectively. Continuing with the same type of repeated measures ANOVA analysis with participant´s mean SJ, CMJ, Pmin, Pmax, YYIR1 and VO_2_max revealed a significant effect of moment, F (2.26) = 7.03, *p* = 0.003, η^2^ = 0.35, F (2.26) = 20.20, *p* = 0.001, η^2^ = 0.60, F (2.26) = 12.41, *p* = 0.001, η^2^ = 0.48, F (2.26) = 8.84, *p* = 0.001, η2 = 0.40, F (2.18) = 10.26, *p* = 0.001, η^2^ = 0.53, and F (2.16) = 9.84, *p* = 0.001, η^2^ = 0.55.

**Figure 2:**
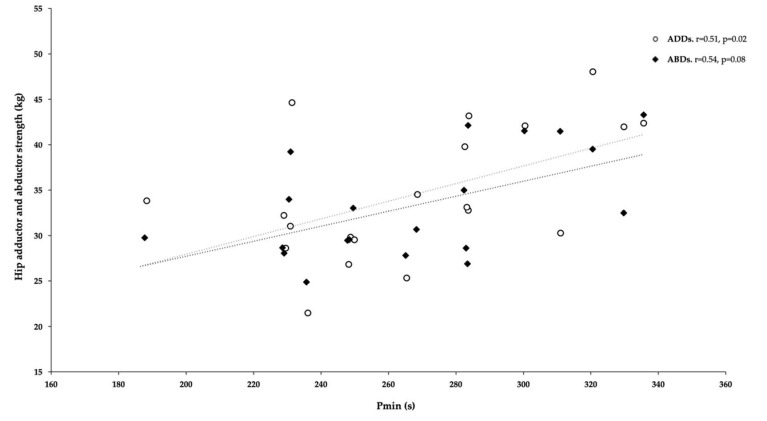
Relationship between hip adductor and abductor isometric strength (ADDs and ABDs) and Pmin of RSA test.

**Figure 3:**
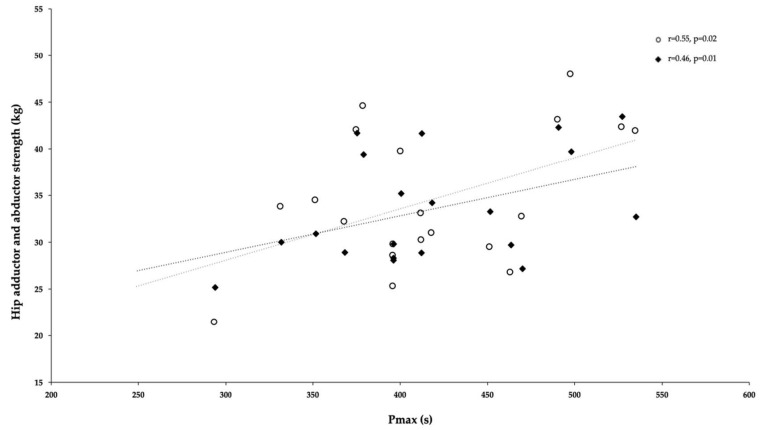
Relationship between hip adductor and abductor isometric strength (ADDs and ABDs) and Pmax of RSA test.

**Figure 4:**
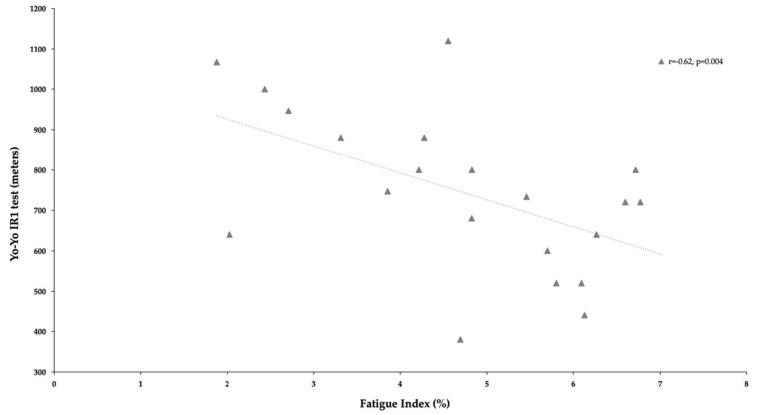
Relationship between Yo-Yo IR1 test and fatigue index (FI) of repeated-sprint ability test.

The correlation coefficients between RSA indices (Pmax, Pmin, and Fatigue index) and fitness variables are summarized in Table 2. No significant correlations were found between all RSA indices and SJ, CMJ, 10m, 30m and COD. However, positive moderate correlations were found between Pmin and ADDs and ABDs [r = 0.51, *p* < 0.02 and r = 0.54, *p* < 0.01, respectively (See Figure 2]. In the same line, positive moderate correlations were found between Pmax and ADDs and ABDs (r = 0.55, *p* < 0.02 and r = 0.46, *p* < 0.01, respectively (see Figure 3)). Last, other interest and negative moderate correlation was found between FI and YYIR1 test [r = −0.62, *p* < 0.004 (Figure 4)].

The regression analysis to predict RSA from physical fitness variables was in agreement with the correlation analysis (See Table 3). On the one hand, ADDs and ABDs were predictor variables of Pmin (r = 0.53 and r = 0.55, respectively). On the other hand, ABDs was predictor variable of Pmax (r = 0.48). Finally, YYIR1 test was a predictor variable of IF (r = −0.53).

## 4. Discussion

The present study aimed to analyze the determinants of RSA based on aerobic performance, linear sprinting and change-of-direction, vertical height jump, and abductor and adductor isometric strength. The main findings were as follows: (i) power in repeated sprints can be improved and predicted through exercises of ABD´s and ADD´s strength, and (ii) RSA can be improved and forecasted through aerobic endurance-based exercises such as the YYIR1 test. Additionally, it was found that RSA, and cardiorespiratory fitness had meaningfully improved over the season, while vertical jump had decreased.

The assessments performed repeatedly over the season revealed a meaningful improvement in RSA and aerobic performance. On the other hand, vertical jump decreased over the season, possibly due to the lack of reactive strength training or oriented training for this physical quality. Usually, both RSA and aerobic performance are key determinants of physical performance in soccer and match running-performance is associated with those capacities [16,46,47], thus it can be expected that over the season the training and match load may explain positive changes in RSA and aerobic performance [48,49]. 

High-intensity efforts, such as sprints, are essential components explaining soccer players´ behavior [50]. However, in addition to sprints in isolation, players perform repeated high-intensity efforts in short intervals, drawing from their aerobic endurance to do so [10]. In this sense, RSA seems to be a suitable method for inducing optimal improvements in anaerobic and aerobic metabolism [18,23,24,25], thus giving a team an advantage over the opponent during moments in matches characterized by high-speed efforts. 

It seems that RSA can be improved through any soccer-specific training program [51], supporting the improvements found in this study over four- and 18-week female soccer training programs. However, the main determinants of RSA test performance among female soccer players are still unclear. Therefore, the authors of the present study tried to analyze the most relevant variables of RSA by comparing indicators from the RSA test (power and fatigue index) with aerobic endurance (YYIR1 test), linear sprinting with COD, vertical height jump (SJ and CMJ test), and ABDs and ADD strength. The primary moderate correlations were established between power and ABD/ADD isometric strength (Pmin and ADD [r = 0.51], Pmin and ABDs [r = 0.54], Pmax and ADDs [r = 0.55], and Pmax and ABDs [r = 0.46]) and between fatigue and YYIR1 test outcomes [r = −0.62]. No other correlations were found between RSA parameters and other tests. 

The relationship between power and ABD/ADD isometric strength may be due to the implication of these muscle groups in sprinting efforts [52]. From an anatomy-based or biomechanic viewpoint, ADD assists hip flexion and neutralizes the abduction and external rotation caused by tensor fascia latae and Sartorius. In addition, during the mid-to-late swing, when the hip is flexed, adductors work as synergists of the gluteus maximus, helping with hip extension and counterbalancing external rotation [52]. On the other hand, the ABDs stabilize the femoral head during high-speed running efforts. They lengthen eccentrically while helping to stabilize the pelvis and control femoral adduction in the transverse plane [53].

Researchers have tried to analyze the implications of hypertrophy of these muscles during sprints. For example, Nuell et al. [52] and Tottori [54] highlighted the implications of and the close relationship between ADD and sprint performance, while Fredericson and Weir [53] highlighted the implications of ABD in gait and sprints. Interestingly, it seems that the implication of ADD correlates with sprinting time [52] and with sprinting distance [54]. These findings encourage physical fitness and conditioning coaches to design coadjutant training programs based on ADD and ABD isometric strength to improve female soccer players’ sprint performance. However, no relationship was found between RSA parameters and exercises with significant quadriceps implications (i.e., SJ, CMJ, and COD).

This idea seems consistent among experts in this topic [55,56], who have concluded that the quadriceps are not related to sprint performance. Instead of the quadriceps, it may be anatomically due to the anterior and middle parts of the gluteus medius, which have a stronger vertical pull and help initiate abduction, which is then completed by the tensor fascia lata [53].

Nevertheless, in addition to strength exercises, aerobic endurance remains crucial during female soccer matches. In the present study, the authors found correlations between fatigue and YYIR1 test results (*p* < 0.004; Figure 4). This finding is supported by Gabrys et al. [57], who concluded that the anaerobic glycolytic system is more sensitive to long, repetitive sprints, highlighting that RSA is a suitable strategy for avoiding insufficient aerobic energy systems, which lead to early decreases in performance [57]. All of these results indicate the value of forecasting Pmax from ADD and ABD isometric strength values (r = 0.53 and r = 0.55, respectively), Pmax from ABD values (r = 0.48), and fatigue from YYIR1 (r = −0.53).

This study had some limitations. The force platforms were not used to calculate the rate of force development during vertical jumps, and this could be interesting. Additionally, an isometric mid-thigh pull test would be interesting to associated with RSA. Future studies should consider analyzing the influence of each physical capacity in different number of sprints, and also consider analyzing COD deficit and asymmetries trying to understand if this can be related with RSA ability. Other limitation is associated with small sample and the specificity of being conducted in women, thus not being possible to generalize for other populations. More research should be conducted to test the replication of results in different scenarios (other competitive contexts, age-groups and populations).

As practical applications, the coadjutant training program—mainly based on these determinants (ABD and ADD isometric strength exercises and YYIR1)—may induce improvements in female soccer players’ RSA and better outcomes during critical moments of matches. Although it was declared that straight sprinting is the most frequent action taken before goals, both for scoring and assisting players [51], the current trend highlights that sprints during soccer games are curvilinear [58,59,60]. As such, they may lead to different demands than straight sprints [57]. Therefore, further studies should assess the main determinants of curvilinear sprinting performance during RSA tests. 

## 5. Conclusions

Power and fatigue are notable RSA-related parameters. Power during RSA is mainly determined by ABD and ADD isometric strength, while fatigue is related to YYIR1. Therefore, physical fitness and conditioning coaches are encouraged to improve ABD and ADD isometric strength alongside aerobic endurance. Doing so may lead to improvements in RSA, subsequently giving the player an advantage over the opponent during critical game situations. However, since it seems that most sprint efforts are made in a curvilinear trajectory, future studies should replicate the present study, focusing on these efforts. 

## Figures and Tables

**Table 1:** Anthropometrical and fitness variables in the three moments of assessment (Mean ± SD).

Women Soccer Players (*n* = 22)							
	Moment 1	Moment 2	Moment 3	CI (95%)	Upper CI (95%)	Lower CI (95%)	Repeated Measures ANOVA (p)
**Hip strength**							
ADDs (kg)		34.66 ± 7.81	35.06 ± 8.12	3.39	37.93	31.15	*p* = 0.97, η^2^ = 0.001.
ABDs (kg)		33.48 ± 5.87	34.19 ± 6.23	2.72	36.14	30.69	*p* = 0.98, η^2^ = 0.001.
**Squat and countermovement jump**							
SJ (cm)	25.33 ± 2.98	26.24 ± 3.09	24.39 ± 3.95	1.38	26.62	23.84	*p* = 0.003 *, η^2^ = 0.35.
CMJ (cm)	27.26 ± 2.98	27.40 ± 3.51	24.65 ± 3.93	1.39	27.70	24.90	*p* = 0.001 *, η^2^ = 0.60.
**Linear sprinting**							
10 m (s)	1.87 ± 0.08	1.90 ± 0.10	1.88 ± 0.10	0.05	1.94	1.85	*p* = 0.26, η^2^ = 0.09.
30 m (s)	4.79 ± 0.23	4.78 ± 0.22	4.75 ± 0.23	0.11	4.90	4.68	*p* = 0.07, η^2^ = 0.16.
**Change-of-direction test**							
COD (s)	5.73 ± 0.19	5.75 ± 0.18	5.79 ± 0.23	0.09	5.86	5.67	*p* = 0.32, η^2^ = 0.08.
**Repeated sprint ability test (RSA test)**							
Pmin (s)	240.44 ± 46.87	267.15 ± 46.29	293.09 ± 36.49	18.29	281.51	244.93	*p* = 0.001 *, η^2^ = 0.48.
Pmax (s)	380.81 ± 68.38	401.77 ± 74.47	444.38 ± 72.96	31.40	441.72	378.92	*p* = 0.001 *, η^2^ = 0.40.
FI (%)	4.61 ± 1.85	4.42 ± 1.66	4.96 ± 1.87	0.70	5.53	4.11	*p* = 0.38, η^2^ = 0.07.
**Yo-Yo intermittent recovery test- Level 1**							
YYIR1. Distance (m)	677.78 ± 203.72	788.00 ± 219.89	863.33 ± 218.73	89.40	833.84	655.04	*p* = 0.001 *, η^2^ = 0.53.
VO_2_max (mL/kg/min)	41.74 ± 5.33	43.02 ± 1.85	43.82 ± 1.82	0.82	43.40	41.75	*p* = 0.001 *, η^2^ = 0.55.

ADD: adductor isometric strength; ABD: abductor isometric strength; SJ: squat jump; CMJ: countermovement jump; 10 m: 10-m sprint; 30 m: 30-m sprint; COD: change-of-direction; YYIRT1: Yo-Yo intermittent recovery test level 1; Pmin: peak power (minimum); Pmax: peak power (maximum); FI: fatigue index; cm: centimeters; s: seconds. * denotes significance at *p* < 0.01.

**Table 2:** Pearson correlation coefficient between RSA indices and fitness variables (*n* = 22).

RSA Indices	ADDs (kg)	ABDs (kg)	SJ (cm)	CMJ (cm)	10m (s)	30m (s)	COD (s)	YYIR1 (m)
**Pmin (s)**	r = 0.51 *p* = 0.02 **	r = 0.54 *p* = 0.01 **	r = 0.13 *p* = 0.58	r = 0.04 *p* = 0.84	R = 0.10 *p* = 0.67	r = 0.09 *p* = 0.70	r = −0.00 *p* = 0.99	r = −0.08 *p* = 0.72
**Pmax (s)**	r = 0.55 *p* = 0.01 **	r = 0.46 *p* = 0.04 *	r = 0.19 *p* = 0.41	r = 0.05 *p* = 0.81	r = −0.10 *p* = 0.65	r = −0.24 *p* = 0.30	r = −0.12 *p* = 0.61	r = −0.38 *p* = 0.10
**FI (%)**	r = 0.33 *p* = 0.16	r = 0.18 *p* = 0.45	r = 0.16 *p* = 0.50	r = 0.03 *p* = 0.87	r = −0.24 *p* = 0.30	r = −0.43 *p* = 0.06	r = −0.17 *p* = 0.47	r = −0.62 *p* = 0.04 *

ADD: adductor isometric strength; ABD: abductor isometric strength; SJ: squat jump; CMJ: countermovement jump; 10 m: 10-m sprint; 30 m: 30-m sprint; COD: change-of-direction; YYIRT1: Yo-Yo intermittent recovery test level 1; Pmin: peak power (minimum); Pmax: peak power (maximum); FI: fatigue index; cm: centimeters; s: seconds. * Denotes significance at *p* < 0.05, and ** denotes significance at *p* < 0.01.

**Table 3:** Values of regression analysis explaining, Pmax, Pmin and Fatigue index based on the remaining variables.

RSA Indices		R	R^2^	Adjusted R^2^	F	P	SE
**Pmin (s)**	**ADDS**	0.53	0.27	0.23	6.86	0.01	34.25
	**ABDs**	0.55	0.30	0.26	8.01	0.01	33.49
**Pmax (s)**	**ADDs**	0.48	0.23	0.19	5.52	0.03	57.60
**FI (%)**	**YYIR1**	−0.53	0.28	0.24	7.39	0.01	1.35

Pmin: peak power (minimum); Pmax: peak power (maximum); FI: fatigue index; s: seconds.
